# Disrupted HSF1 regulation in normal and exceptional brain aging

**DOI:** 10.1007/s10522-023-10063-w

**Published:** 2023-09-14

**Authors:** Rachana Trivedi, Bailey Knopf, Sharlene Rakoczy, Gunjan D. Manocha, Holly Brown-Borg, Donald A. Jurivich

**Affiliations:** 1 Department of Geriatrics, School of Medicine and Health Sciences, University of North Dakota, 1301 N Columbia Rd, Grand Forks, ND 58201 USA; 2 Department of Biomedical Sciences, School of Medicine and Health Sciences, University of North Dakota, Grand Forks, ND 58202 USA

**Keywords:** Heat shock, Brain aging, Longevity, HSF1, Ames Dwarf

## Abstract

Brain aging is a major risk factor for cognitive diseases such as Alzheimer’s disease (AD) and vascular dementia. The rate of aging and age-related pathology are modulated by stress responses and repair pathways that gradually decline with age. However, recent reports indicate that exceptional longevity sustains and may even enhance the stress response. Whether normal and exceptional aging result in either attenuated or enhanced stress responses across all organs is unknown. This question arises from our understanding that biological age differs from chronological age and evidence that the rate of aging varies between organs. Thus, stress responses may differ between organs and depend upon regenerative capacity and ability to manage damaged proteins and proteotoxicity. To answer these questions, we assessed age-dependent changes in brain stress responses with normally aged wild type and long-lived Dwarf mice. Results from this study show that normal aging unfavorably impacts activation of the brain heat shock (HS) axis with key changes noted in the transcription factor, HSF1, and its regulation. Exceptional aging appears to preserve and strengthen many elements of HSF1 activation in the brain. These results support the possibility that reconstitution of aging brain stress responses requires a multi-factorial approach that addresses HSF1 protein levels, its DNA binding, and regulatory elements such as phosphorylation and protein interactions.

**Supplementary Information:**

The online version contains supplementary material available at 10.1007/s10522-023-10063-w.

## Introduction

Aging is a complex process with accumulation of damaged and misfolded proteins that lead to reduced cellular viability and the occurrence of protein misfolding diseases such as Alzheimer’s dementia (Taylor and Dillin 2011; Krisko and Radman 2019). In order to survive hostile environments induced by disease, inflammation, and proteotoxicity, cells must be able to defend themselves rapidly and decisively (Mukhopadhyay et al. 2003). Inducible stress pathways such as the heat shock response are an essential mechanism used by cells to protect themselves from potentially damaging insults (Richter et al. 2010). Upregulated expression of chaperones and heat shock proteins (HSPs) from their baseline levels help prevent damage and aggregation at the proteome level (Le Breton and Mayer 2016). While all organs and tissue have heat shock proteins, emerging evidence suggests that the heat shock response is not uniform across all tissue and their cells (Carnemolla et al. 2015; Ma et al. 2017). Furthermore, aging may exaggerate differences in various tissue-specific heat shock responses especially given that organs appear to age at different rates over the lifespan (Jazwinski and Kim 2019; Walker and Herndon 2010; Trivedi and Jurivich 2020).

In the context of the role that HSF1 plays in neuroprotection, several studies reveal the central importance of this transcription factor in neuronal survival and maintenance of neuronal health by countering the accumulation of toxic protein aggregates (Calderwood and Murshid 2017; Gomez-Pastor et al. 2017). Conversely, dysregulated HSF1 has been linked to many age-related neurodegenerative diseases including Alzheimer’s and Huntington’s Disease. In preclinical Alzheimer’s Disease models, HSF1, HSP70 and HSP90 proteins are expressed at low levels, a finding that recapitulates normal aging (Jiang et al. 2013). Total HSF1 protein in brain samples exhibit a significant decline in brains of mouse AD models and in postmortem human Huntington’s Disease patients (Gomez-Pastor et al. 2017). When normal neurons treated with a compound causing neuronal death, HSF1 mRNA and protein levels decline long before signs of neuronal degradation are detected (Verma et al. 2014). These studies suggest that changes in HSF1 levels and its regulation play a crucial role in neuroprotection and neurodegeneration.

An additional question arises whether exceptional longevity preserves the aging stress response particularly as it relates to the brain. If so, are there unique features of the heat shock axis that can be emulated to deter usual aging processes (Le Breton and Mayer 2016)? This inquiry brings an additional interest in how biological age rather than chronological age impacts the stress response, perhaps accounting for the variability observed in heat shock response within an aging cohort. To address these questions, we examined the regulatory checkpoints of the HS axis in brain tissue extracted from 3 M, 12 M and 22 M old Ames Dwarf and wild type mice with the hypothesis that exceptionally long-lived mice would exhibit less heat shock attenuation than age-matched wild type mice. HSF1 has a series of steps associated with its activation including trimer formation for DNA binding, deacetylation, phosphorylation and disassociation from regulatory proteins. Thus, this investigation examined several regulatory checkpoints of HSF1 activation across the mouse lifespan. In parallel with usual aged mice, we examined whether exceptionally long lived mice sustained the HS response during their life span or possibly had an overall enhanced response.

## Material and methods

### Antibodies and reagents

Anti-HSF1 (51,034-1-AP), anti-HSP90 (13,171-1-AP), anti-HSP70 (10,995-1-AP), anti-GAPDH (10,494-1-AP) and anti- HSP40 (13,174-1-AP) were rodent specific and were all purchased from ProteinTech Group while Phospho-HSF1 (Ser303) Polyclonal Antibody (PA5-105,806), Phospho-HSF1 (Ser307) Polyclonal Antibody (PA5-105,806) and Phospho-HSF1 (Ser326) Polyclonal Antibody (BS-3741R), and anti-FBXW7 (40-1500) were purchased from ThermoFisher Scientific. Anti-SIRT1 (3931S), anti-p38 (8690S), and Phospho-p38 (4511S) were purchased from Cell Signaling Technologies. The horseradish peroxidase-conjugated secondary antibodies were purchased from BioRad Laboratories Inc. Primers for HSF1 (F-ACAGTGTCACCCGGCTGTTG and R-GACTGCACCAGTGAGATGAGGAA), HSP70 (F-CATGGTGGTTGCACTGTAGG and R-ATTGACCCGAGTTCAGGATG), HSP40 (F-CTCCAGTCACCCATGACCTT and R-CGCTTGTGGGAGATTTTCAT), HSP90 (F-CAATGACTGGGAGGACCACT and R-CAATGCCCTGAATTCCAACT) and GAPDH (F-AACTTTGGCATTGTGGAAGG and R-GGATGCAGGGATGATGTTCT) were purchased from Eurofins MWG Genomics LLC and HSE probe from Integrated DNA Technologies.

### Animals

To study the effects of aging on HSF1 regulation, we studied 3, 12 and 22-month-old male Ames Prop1^df/df^ Dwarf and wild-type mice brains. Smaller but parallel experiments were done with female mice to establish gender differences, any, in the assays used to dissect the age dependent signaling pathways of HSF1 activation. Ames Dwarf and wild-type mice were bred and maintained at the Center for Biomedical Research, University of North Dakota, under controlled pathogen-free conditions of photoperiod 12:12-h light/dark cycles and temperature (22 ± 1 °C) with ad libitum access to food and water. These mice have a heterogeneous genetic background from a colony that has been closed for over 30 years. The investigation conforms to the National Research Council of the National Academies Guide for the Care and Use of Laboratory Animals (8th edition) and was reviewed and approved by the UND IACUC. Ames wild-type and Dwarf mice ages 3 M, 12 M and 22 M were sacrificed by CO_2_ asphyxiation followed by de-capitation. Brain tissue from 5–8 mice per group was isolated and lysed using RIPA and protein was quantitated using Pierce™ Rapid Gold BCA Protein Assay Kit.

### Electrophoretic mobility shift assay (EMSA)

EMSAs were performed using the LightShift Chemiluminescent EMSA Kit (Active Motif) according to the manufacturer’s protocol. Whole cell extracts from brain tissue were prepared in buffer C (20 mM HEPES, pH 7.9, 25% Glycerol, 0.42 M NaCl, 1.5 mM MgCl2, 0.2 mM EDTA, 0.5 mM PMSF, and 0.5 mM DTT), and 15 µg protein was incubated with 1 µg poly[d(I-C)], 2 µl of 10X binding buffer, 1 µl of 50% glycerol, 1 µl of 1% NP-40, 1 µl of 100 mM MgCl2 and 20 fmol of biotin end-labeled probe (HSE biotinylated probe CTAGAAGCTTCTAGAAGCTTCTAG) in a = total volume of 20 µl for 30 min at 25 ℃. After separation by 5% TBE polyacrylamide gel electrophoresis, the protein-DNA complexes were transferred to a nylon membrane (Pierce) and the membranes were UV-cross linked for 10 min. The biotin labeled DNA was probed with streptavidin-HRP conjugate for chemiluminescence detection. The band corresponding to the HSF1-HSE complex was quantitated for Optical Density measurement and plotted ± SEM (n = 3–5).

### Western blotting

20 µg brain lysate from both Ames wild type and Ames Dwarf mice were resolved by SDS-PAGE and transferred to polyvinylidene difluoride membranes for Western blotting using anti-HSF1 (1:1,500), anti-HSP40 (1:2,000), anti-HSP70 (1:2,000), anti-HSP90 (1:2,000), anti-HSF1 Ser303 (1:500), anti-HSF1 Ser307 (1:500), anti-HSF1 Ser326 (1:500), anti-SIRT1 (1:1,000), anti-p38 (1:500), anti-pp38 (1:500), anti-FBXW7 (1:500) and anti-GAPDH (1:10,000; loading control) antibodies. Antibody binding was detected using enhanced chemiluminescence for detection (Omega Lum G Aplegen). Western blots were quantified using UltraQuant software for densitometry. Optical density (O.D.) of bands were normalized against their respective loading controls and averaged (± SEM).

### Immunohistochemistry

A half cut paraformaldehyde fixed brain from 3 M, 12 M, and 22 M old both WT and Dwarf mice were cut using a temperature controlled cryostat. Briefly, paraformaldehyde-fixed tissue was embedded in a 15% gelatin (in 0.1 M phosphate buffer) matrix and immersed in a 4% paraformaldehyde solution for 2 days to harden the gelatin matrix. The blocks were then cryoprotected through three cycles of 30% sucrose for 3–4 days each. The blocks were then flash frozen using dry-ice/isomethylpentane, and 40µ serial sections were cut using a cryostat at – 17 °C. Serial sections were immunostained using anti-HSF1 antibody 1:250, followed by incubation with biotinylated secondary antibody and avidin/biotin solution (Vector ABC kit). Immunoreactivity was visualized using Vector VIP as chromogen. The slides were dehydrated and coverslipped using VectaMount (Vector Laboratories) following a standard dehydrating procedure through a series of ethanol concentrations and Histo-Clear (National Diagnostics). Images were taken using an upright Leica DM1000 microscope and Leica DF320 digital camera system.

### Two-dimensional gel electrophoresis

Two-dimensional gel electrophoresis was performed by Kendrick's Labs Inc. (Madison, WI) according to the carrier ampholine method of isoelectric focusing. Isoelectric focusing was carried out in a glass tube of inner diameter 3.3 mm using 2.0% pH 3–10 Isodalt mix Servalytes (Serva, Heidelberg, Germany) for 20,000 V-hours. One microgram of an IEF internal standard tropomyosin was added to each sample. This protein migrates as a doublet with lower polypeptide spot of molecular weight 33,000 Da and pI 5.2. The enclosed tube gel pH gradient plot for this set of Servalytes was determined with a surface pH electrode.

After equilibration for 10 min in Buffer “O” (10% glycerol, 50 mM dithiothreitol, 2.3% sodium dodecyl sulfate, and 0.0625 M tris, pH 6.8), each tube gel was sealed to the top of a stacking gel that overlaid a 10% acrylamide slab gel (1.00 mm thick). Sodium dodecyl sulfate slab gel electrophoresis was carried out for about 5 h at 25 mA per gel. After slab gel electrophoresis, the gels were placed in transfer buffer (10 mM CAPS, pH 11.0, 10% MeOH) and transblotted onto polyvinylidene difluoride (PVDF) membranes overnight at 225 mA and approximately 100 V per two gels. The following proteins (Millipore Sigma) were used as molecular weight standards: myosin (220,000 Da), phosphorylase A (94,000 Da), catalase (60,000 Da), actin (43,000 Da), carbonic anhydrase (29,000 Da), and lysozyme (14,000 Da). These standards appear as bands at the basic edge of the Coomassie Brilliant Blue R-250-stained membrane.

The blots were stained with Coomassie Brilliant Blue R-250 and desktop scanned. The blots were wetted in 100% methanol, rinsed briefly in Tween-20 tris buffered saline (TTBS), and blocked for 2 h in 5% bovine serum albumin in TTBS. The blots were then incubated in primary antibody (anti-acetylated lysine [Cell Signaling, Cat no. 9441, Lot no. 11] diluted 1:10,000 in 2% bovine serum albumin in TTBS) overnight and rinsed 3 × 10 min in TTBS. The blots were then placed in secondary antibody (anti-rabbit IgG HRP [Sera Care, Cat no. 5220–0337, Lot no. 10245261] diluted 1:20,000 in 2% bovine serum albumin in TTBS) for 2 h, rinsed in TTBS as earlier, treated with enhanced chemiluminescence, and exposed to x-ray film.

### Real-time PCR

RNA was extracted from brain tissue of 3 M, 12 M and 22 M old Ames wild type and Dwarf mice using RNeasy Mini kit (Qiagen). cDNA was prepared using RevertAid First Strand cDNA Synthesis kit (ThermoScientific) and real time PCR for HSF1 HSP40, HSP70 and HSP90 was performed with the housekeeping gene, GAPDH, using the Biorad CFX Connect System. Fold change in mRNA expression is represented as 2^ΔΔ-C_t_ SEM (n = 10–14).

### Statistical analysis

Data are presented as bar graphs which represent the mean ± SEM. Values statistically different from controls were determined using one-way ANOVA (or two-way ANOVA where required). The Tukey–Kramer multiple comparisons post-test was used to determine p-values.

## Results

### HSF1 DNA binding, protein levels and mRNA levels:

**Fig. 1:**
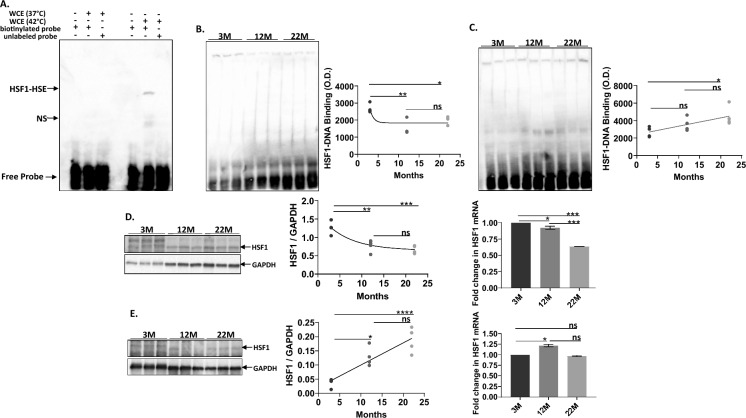
Graph representation of age – dependent HSF1 changes (DNA binding, protein and mRNA levels) in wild type and long-lived mice brain tissue.** A** shows a standardized Electromobility shift assay (EMSA) for HSF1-HSE binding in HeLa cell extracts under normal and heat shock conditions. HeLa cells under normal conditions (37 °C) exhibit no HSF1-HSE binding and cells exposed to 42 °C for 30 min exhibit increased HSF1-HSE binding. HSF1-HSE binding specificity is demonstrated as excess unlabeled DNA probe blocks HSF1-HSE but not nonspecific protein-HSE binding. **B **and** C** show EMSA of HSF1–HSE binding in brain tissue extracts from male wild type and Dwarf mice respectively at ages 3 M, 12 M and 22 M (n = 5 per age cohort). Optical density measurement of the HSF1–HSE band shows an age-dependent decline in HSF1– HSE binding activity (p = 0.047) in wild type brain tissue (**1B**) and increased HSF1-HSE binding in aging Ames Dwarf mice (n = 10, p = 0.0366) (**1C**) (p = 0.0405). **D** shows that both HSF1 protein levels (p = 0.0005) and mRNA (p = 0.0002) decline in aging wild type brain; whereas, **E** shows that HSF1 protein levels increase with age in the long lived mice (p = 0.0332) while HSF1 mRNA levels (p = 0.3479) do not change over time. HSF1 protein levels are standardized to GAPDH levels. *HSE* heat shock element, *HSF* heat shock factor, *NS* non specific protein binding, *O.D.* Optical density, *WCE* whole cell extract

One of the first and well characterized steps in HSF1 activation is the assembly of monomers into trimeric units capable of binding to nGAAn repeats in the heat shock promoter (Hentze et al. 2016). Therefore, we examined the age-dependent changes in HSF1–DNA binding activity in both wild type and long lived Dwarf mice using the Electromobility Shift Assay (EMSA). HSF1–DNA binding from freshly isolated brain tissue from 3 M, 12 M and 22 M old male wild type and Dwarf mice is shown in Fig. 1B and C. Female brain extracts showed the same age-dependent decline in HSF1–DNA binding (data not shown). Figure 1B shows representative samples from aging wild type mice whereby HSF1–DNA binding declines by 25% between 3 and 12 M old brain samples with sustained age-dependent loss of HSF1-DNA binding in the 22 M mice (p = 0.0473, 0.2960). By contrast, brain tissue from long-lived 22 M (n = 6) Ames Dwarf mice exhibit progressively higher HSF1-DNA binding with each age group, as shown in Fig. 1C. Compared to 3 M brain samples, the 12 and 22 M old brains manifest 25 to 67% stronger HSF1-DNA binding (p = 0.3243, 0.0366). Thus, age enhances HSF1-DNA binding in Dwarf but not wild type brain tissue.

Given the loss of HSF-DNA binding with usual aging and its gain during exceptional aging (Jurivich et al. 2020; Trivedi et al. 2021) we examined HSF1 protein and mRNA levels between the two aging groups of mice. Figure 1D shows that HSF1 protein levels at 3 M steadily decline in 12 M and 22 M old wild type brain by 47% and another 9% respectively (p = 0.005 for 3 vs 12 M and p = 0.5029 for 12 vs 22 M). Quite opposite findings with age are revealed in Dwarf brain samples. As seen in Fig. 1E, HSF1 protein levels progressively increase from 3 to 12 M to 22 M, representing a 397% increase when 3 M are compared to 22 M Dwarf brains (p = 0.004).

To investigate whether the change in HSF1 protein levels were reflected at the mRNA level, real time PCR was used to evaluate each of the WT and Dwarf age groups with fold changes shown in Fig. 1D and E. Similar to the loss of HSF1 protein levels with age in wild type mice, HSF1 mRNA declined with age by 36%, although the loss was most significant going from the 12 M to the 22 M old age group (p = 0.0005). HSF1 mRNA in Dwarf brain did not appear to change significantly with age, albeit the 12 M samples exhibited a modest bump in HSF1 mRNA levels relative to the two other age groups (p = 0.0018).

To further corroborate age-dependent changes in HSF1 protein levels, we evaluated HSF1 protein expression using immunohistochemistry in 3 M, 12 M, and 22 M hippocampal sections from both wild type and Dwarf mice. eFigure 1A in the Supplement confirms our Western Blot analysis that that HSF1 protein levels decreased in 22 M old wild–type mice as compared to 12 M old mice brain tissue. eFigure 1B in the Supplement further confirms the age—related accumulation of HSF1 protein in Dwarf hippocampus.

### Regulatory HSPs

**Fig. 2:**
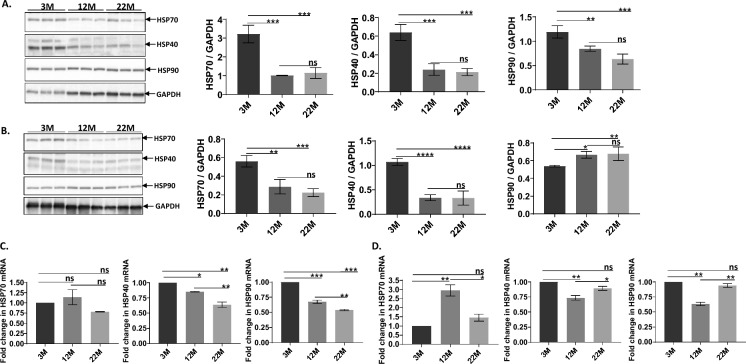
Bar graphs represent age-dependent changes in brain heat shock proteins that regulate HSF1 among wild type and Dwarf mice. **A** shows declining protein levels of HSP70, HSP40 and HSP90 in aging wild type brain (n = 5, p = 0.0005, p = 0.0005, p = 0.0005) with **B** showing a similar age-dependent downward trend in Dwarf brain HSP70 and HSP40 levels albeit HSP90 protein levels increase in mid to late life (p = 0.0332). **C **and** D** show age-dependent reduction in mRNA for HSP70, HSP40 and HSP90 in both wild type (p = 0.2466, p = 0.0016 and p = 0.0002) and Dwarf brain (p = 0.2175, p = 0.0726 and p = 0.1752) although the values in Dwarf aging brain were not statistically significant

Another regulatory HSF1 checkpoint examined was the status of heat shock proteins known to interact with and regulate HSF1 transcriptional activity (Shi et al. 1998; Masser et al. 2020; Gomez-Pastor et al. 2018). HSP40, HSP70 and HSP90 protein levels in both wild type and Dwarf mice were evaluated by Western Blots. Figure 2A shows Western Blots and bar graphs that demonstrate all three regulatory heat shock protein levels decline between 50 and 75% within aging wild type brain, commencing at 12 M of age and sustained through 22 M of age. Figure 2B graphically shows that Dwarf mice brain also exhibit a decline in HSP40 and HSP70 protein levels with age after 3 M, but HSP90 levels increase in the 12 M and 22 M age groups. Relative to 3 M, HSP90 levels increase in 22 M old Dwarf brain by 25%.

HSP mRNA levels were assessed by real time PCR in the three different age groups of WT and Dwarf mice. Similar to loss of HSP protein levels with age, Fig. 2C shows that mRNA levels decline with age in WT brain for each HSP with the caveat that HSP70 mRNA levels trended downward but not significantly in 22 M old brain. Figure 2D shows mRNA levels for the HSP in Dwarf brain. When comparing 3 M with 22 M Dwarf brain samples there is no statistically significant difference in each of the HSP mRNAs. The 12 M Dwarf brain samples exhibit mixed results for age-dependent HSP mRNA levels, demonstrating either an increase for HSP70 mRNA relative to 3 M samples or a decrease for HSP40 and HSP90 mRNA.

### HSF1 posttranslational modifications:

**Fig. 3:**
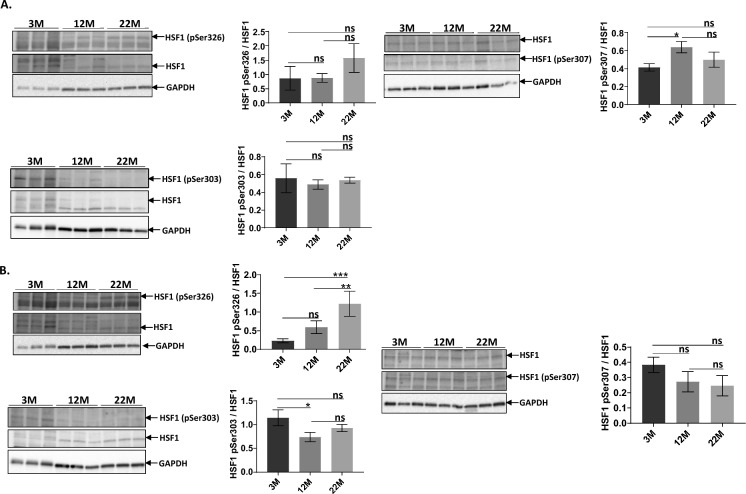
Western blot and graph analysis of age-dependent changes in activating and deactivating phosphorylation of HSF1 between different age groups of Ames wild type and Dwarf mice brain.** A** shows western blot analysis of HSF1 phosphorylation levels at serine residues 326 (HSF1 activating) and 303/307 (HSF1 deactivating) accompanied by a bar graph display of HSF1 phosphorylation levels normalized to HSF1 protein levels in wild type mice. **B** shows that activating HSF1 phosphorylation (Ser326) significantly increases with age in Dwarf mice while inhibitory phosphorylation at Ser303/307 declines with age in an insignificant manner. Data shown are as the mean ± SEM. *ns* not significant, *P* > 0.05; *P < 0.05; **P < 0.001; ***P = 0.0001; ****P < 0.0001

HSF1 trans-activating properties are partly regulated by its phosphorylation (Hietakangas et al. 2003; Xia and Voellmy 1997), thus we assessed the pro-activation of HSF1 phosphorylation at Ser326 and the inhibitory regulation at Ser303/307. Figure 3A shows both a Western Blot and bar graph quantification of the three different phosphorserine levels for each age group. These values are normalized to HSF1 protein levels to accommodate the age – dependent changes in HSF1 protein. In wild type brain, neither the levels of pro-activating phosphorylation at Ser326 residue or de-activating phosphorylation at Ser303/307 change with age when normalized against HSF1. By contrast, Dwarf brain showed a significant increase with age in pSer326 residues (p = 0.0003) as seen in Fig. 3B. Deactivating phosphorylation levels of HSF1 at Ser303 decreased at 12 M (p = 0.0108) and 22 M Dwarf mice (p = 0.1465), noting the latter value did not achieve statistical significance. Deactivating phosphorylation of HSF1 at Ser307 declined in an age-dependent manner in Dwarf brain samples when normalized against HSF1. As a reference point, similar trends in HSF1 phosphorylation changes, or lack of changes, were observed when HSF1 phosphorylation levels were normalized to GAPDH (data not shown).

**Fig. 4:**
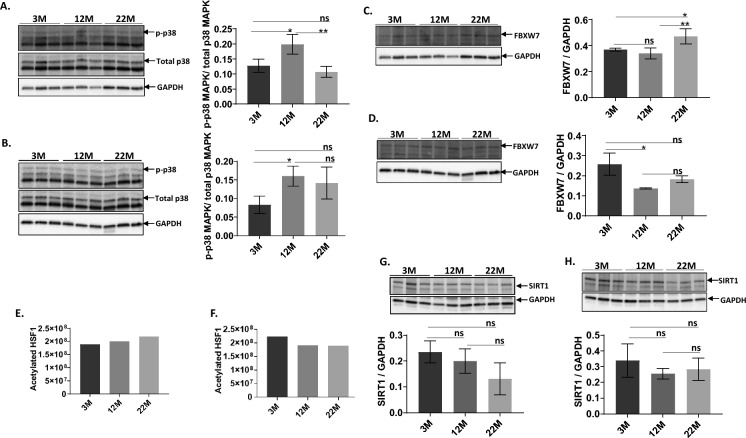
Western blot, HSF1 Acetylation, and graph analysis of age-dependent changes in representative protein kinases that regulate HSF1 phosphorylation, p38 MAPK kinase, Fbxw7 and SIRT1. **A **and** C** show that age impacts p38 MAPK kinase and Fbxw7 protein levels in wild type brain. p38 MAPK kinase levels increase from 3 to 12 M cohorts but decline from 12 to 22 M groups; whereas, Fbxw7 protein levels increase only in the 22 M old cohort. By comparison, **B **and** D** show that p38 MAPK and Fbxw7 levels do not appreciably change with age in Dwarf mice. Data shown are as the mean values + SEM. Where n.s. = not statistically significant based on *P* > 0.05 and values of significance = *P < 0.05; **P < 0.001. Age-dependent changes in HSF1 acetylation are shown in **E **and** F** bar graphs. **G **and** H** show Western blot and bar graph analysis of SIRT1 protein levels in 3 M, 12 M and 22 M old hippocampi of wild type and Dwarf mice respectively. **G** shows a progressive 44% decrease in in SIRT1 protein levels over the life span of  aging Ames wild type mice (*p* = 0.1028); whereas, an age - dependent decrease in SIRT1 protein levels was only 14% (*p* = 0.6683) in Ames Dwarf mice **H**

Changes noted in HSF1 phosphorylation status with age raised questions about protein kinases that regulate HSF1 such as p38 MAPK which controls HSF1 activating Ser326 phosphorylation (Dayalan Naidu et al. 2016). Figure 4A shows a Western blot analysis of p38 MAPK from brain tissue in 3 M, 12 M and 22 M wild type mice. An accompanying bar graph shows that p38 MAPK declines with age (12 M vs 22 M, p = 0.0066). In contrast, Fig. 4B shows analysis of p38 MAPK levels in Dwarf brain which increase with age at the 12 M juncture (p = 0.0331).

HSF1 ubiquitination is another downregulatory process for the HS axis via Ser303/307 mediated proteasomal degradation of HSF1 (Gomez-Pastor et al. 2017), which may play a role in brain aging. Thus, we examined protein levels of the HSF1 ubiquitin ligase FBXW7 with age. Western blot analysis shows that FBXW7 levels significantly increases with age (vs 3 M p = 0.0235, vs 12 M p = 0.0063) in wild type brain tissue (Fig. 4C). However, as seen in Fig. 4D, FBXW7 expression is downregulated with age (p = 0.0180) in Dwarf brain.

Given that HSF1 acetylation impairs HSF1-DNA binding activity, two-dimensional gel electrophoresis followed by Western blot analysis of whole cell lysate from 3 M, 12 M and 22 M old brain hippocampus HSF1 acetylation was done. eFigure 2A in the Supplement shows overall protein acetylation and acetylated HSF1 in 3 M, 12 M and 22 M old brain hippocampus from Ames wild type and Dwarf mice. Bar graphs generated from scanning densitometry of the Western blots shows that HSF1 acetylation increases with age in Ames wild type mice (Fig. 4E) and decreases in Dwarf mice brain hippocampus (Fig. 4F). eTable 1 in the Supplement summaries densitometry values for the various isoforms of acetylated HSF1 proteins. Of the 39 HSF1 isoforms, 46% show an increased level of acetylation in normal 3 M to 12 M hippocampal aging, whereas 43% of isoforms increased acetylation in 3 M compared to 22 M (increase > 25%). On the contrary, HSF1 isoforms in Ames Dwarf hippocampi show a 42% decrease in acetylation from 3 to 12 M and only a 23% decrease between 3 and 22 M (decrease > 25%).

Having noted critical changes in HSF1 acetylation with aging and longevity, we examined Sirtuin 1 (SIRT1), a nicotinamide adenosine dinucleotide (NAD)-dependent deacetylase, that tightly regulates several stress-induced transcription factors including p53, NF-kB, PGC-1α, HSF1 and FOXO family of transcription factors (Gong et al. 2014). SIRT1 regulates the heat shock response through deacetylation of HSF1-DNA binding domain and facilitate HSF1 binding to DNA. Therefore, we evaluated the change in SIRT1 protein levels in brain hippocampus from 3 M, 12 M and 22 M old Ames wild type and Dwarf mice. Figure 4G and H demonstrates the age-dependent changes in SIRT1 protein level in brain hippocampus from Ames wild type and Dwarf mice. Bar graph of densitometry values for SIRT1 Western Blots from Ames wild type hippocampi shows that SIRT1 levels decline with age (Fig. 4G; 3 M vs 22 M, p = 0.1028) while SIRT1 levels in 22 M Dwarf brain hippocampi show a relatively slighter decline in SIRT1 level when compared to 3 M old hippocampi (Fig. 4H; p = 0.6683).

## Discussion

HSF1 is strongly linked to longevity and possibly disease resistance. It’s down regulation accelerates tissue aging and shortens life span in *C. elegans* (Hsu et al. 2003). Another study shows that brain and astrocytes from HSF1^−/−^ knockout mice increases ubiquitinated proteins and sensitivity to oxidative stress (Akerfelt et al. 2010). Suboptimal stress responses are found in neurodegeneration (Neef et al. 2011). Overall, HSF1 is implicated in the quality control and maintenance of CNS homeostasis. Its loss of function with age may represent the concept of homeostenosis or loss of resiliency.

Conversely, HSF1 over expression increases absolute life spans which is considered a prerequisite for healthy lifespans (Morley and Morimoto 2004). HSF1 provides neuroprotection by regulating the transcription of genes encoding heat shock proteins (Verma et al. 2014; Qu et al. 2018). This effect entails trimeric HSF1 binding to heat shock gene promoters that increase transcription of mRNA for heat shock proteins. A challenge to this canonical view emerged from a recent study that describes a mutated form of HSF1 incapable of either homotrimerization or stimulation of HSP mRNA expression and yet these neurons survive (Qu et al. 2018). The exact role of HSF1 isomers and its multimeric state remain to be seen (Neef et al. 2010; Fujimoto et al. 2005).

An emerging concept is that HSF1 may serve in a non-transcriptional role and directly mediate cell protection. HSF1 activators such as HSF1A inhibit polyQ protein aggregation and mitigate its cytotoxic effects (Neef et al. 2010). A preclinical model of Huntington’s disease demonstrated that constitutively active HSF1 reduces polyglutamine aggregation (Fujimoto et al. 2005). HSF1 neutralizes soluble amyloid oligomers (AOs) via physical interactions and protects the essential mitochondrial chaperone HSP60 from degradation (Tang et al. 2020). Dominant—positive HSF1 inhibits alpha-synuclein aggregation and toxicity in SH-S5SY cells (Kim et al. 2016). Disassociation of HSF1 and HSP90 with geldanamycin improves cell viability in a Drosophila model of Parkinson’s disease) Auluck et al. 2005; Auluck and Bonini 2002; Neef et al. 2010).

Taken together, these studies implicate HSF1 in neurodegenerative processes and further raise the issue of how age influences HSF1 activation as a segue to age-dependent diseases. Previous research on the aging stress response reveals defective HSF1 activation and disrupted signaling that attenuates HSF1 (Trivedi et al. 2021; Jurivich et al. 2020). Furthermore, emerging evidence suggests that longevity is associated with either preservation of HSF1 activation or its enhancement over time (Trivedi et al. 2021).

### HSF1 DNA binding activity and protein levels

**Fig. 5:**
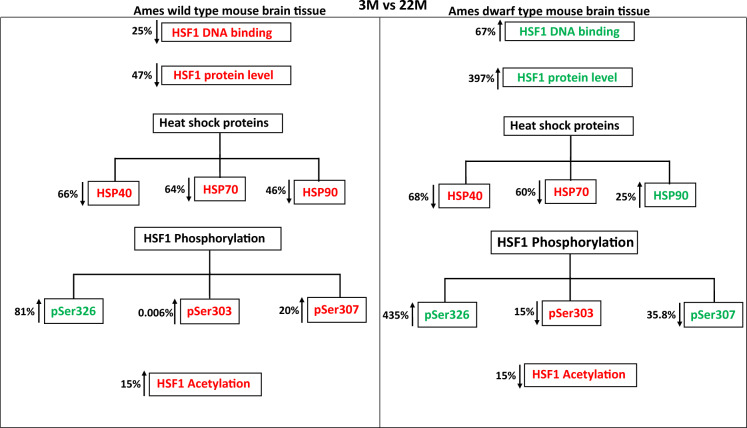
Differences in HSF1 checkpoints during brain aging of both Ames wild type and Dwarf mice

This study shows important differences of HSF1 checkpoints during brain aging in wild type and long lived mice (Fig. 5). Consistent with other studies on aging, HSF1–DNA binding levels in brain from wild type older mice decrease from 30 to 50% relative to brains from younger mice (3 M). Unlike other studies, we find that the age-dependent decline in brain HSF1 – DNA binding occurs at the 12 M juncture rather than the 20–24 M period of time in other mouse tissue (Trivedi et al. 2021). By contrast, brain samples from long lived Dwarf mice demonstrate a steady increase in HSF1-DNA binding over the life span. The surprising observation that HSF1–DNA binding escalates with age in long lived mice can be interpreted in several ways. HSF1 is a known longevity factor and it may become more active over the life span of Dwarf mice to fend off age-dependent damage and manage proteostasis as a requirement of long life. Alternatively, elevated HSF1–DNA binding in long lived mice may drive longevity pathways in addition to the heat shock axis, albeit no evidence exists for longevity genes. HSF1 is known to transactivate non-heat shock genes which may play a role in longevity*.* Unknown is whether the linear increase in HSF1-DNA binding over the Dwarf life span is a compensatory mechanism driven by increasing proteotoxicty with age or due to other stimuli.

As HSF1-DNA binding levels decline with normal aging, so do HSF1 protein levels. By contrast, HSF1 protein levels increase in long lived mice. These data, for the first time, implicate HSF1 protein levels as being important to the integrity of HSF1–DNA binding with age. During normal aging, HSF1 mRNA levels decline but not in Dwarf mice. This observation suggests that HSF1 protein turnover in Dwarf mice is slower than in wild type mice. These data raise the question of optimal HSF1 protein levels required to sustain HS responses throughout the lifespan. Longevity enhancement of brain HSF1 activation is also seen in liver tissue from Dwarf mice (Trivedi et al. 2021), thus suggesting that a stable or enhanced heat shock axis is a prerequisite to exceptional longevity.

### Post translational changes in HSF1

Checkpoints of HSF1 activation that involve posttranslational modifications such as phosphorylation and acetylation are changed during the lifespan of wild type and Dwarf mice. Normal and exceptional brain aging both lead to increased pSer326 residues in HSF1. Increased pSer326 levels implicate changes associated with p38 MAPK activation. Previous studies show that p38 MAPK causes HSF1 phosphorylation at Ser326 and transcriptionally activates HSF1 (Dayalan Naidu et al. 2016; Guettouche et al. 2005). Thus, elevated protein levels of p38 MAPK in Dwarf brain substantiates the idea that exceptional longevity accentuates HSF1 activation over and beyond that observed in normal aging mice. We interpret the elevated pSer326 in normal aged mice as a result of decreased phosphatase activity as they did not show changes in p38 MAPK levels.

With regard to the negative regulation of HSF1 with age via phosphorylation of Ser303 and Ser307, we observed mixed results with no obvious trends in negatively regulating phosphoserine residues with either usual aging or longevity. These measurements differ from aging liver tissue where pSer303 and pSer307 levels increase with age (Trivedi et al. 2021). These data suggest tissue differences in the signaling pathways that may negatively affect HSF1 during senescence.

Another key regulator of HSF1 transcriptional activity is acetylation of the conserved lysine in position 80 (K80) residue. K80 acetylation blocks HSF1–DNA binding activity (Zelin and Freeman 2015). Previously, we showed markedly increased HSF1 acetylation in liver samples from aging wild type mice and decreased HSF1 acetylation in Ames Dwarf mice liver (Trivedi et al. 2021). In a similar fashion, we found an age-dependent increase in HSF1 acetylation in Ames wild type hippocampus and a decrease in HSF1 acetylation in Ames Dwarf hippocampus. Interestingly, our data show that the increase in HSF1 acetylation during normal aging occurs around 12 M and is maintained throughout the lifespan. To our knowledge, this is the first study to show altered HSF1 acetylation starting around the 12 M time period in Ames wild type mice, thus providing a clue as to when anti-aging interventions may need to start for reconstituting the aging stress response. While several mechanisms can reduce HSF1 affinity for DNA, the acetylation of K80 is perhaps the most plausible explanation for age-dependent loss of HSF1-DNA binding activity with age. The loss of HSF1 protein with age may also be linked to reduced deacetylase activity with age as a recent study shows a connection between SIRT1 activity and maintenance of HSF1 protein levels that are otherwise degraded by a NEDD4-mediated pathway (Kim et al. 2016). Thus, our results implicate the acetylation status of the Lys 80 residue located in the DNA-binding domain of HSF1 as a critical factor in modulating HSF1 protein stability in addition to its previously identified role in the transcriptional activity.

The relationship between SIRT1 and age is well established, and various studies show that caloric restriction extends life by enhancing SIRT1 expression (Canto and Auwerx 2009; Lee et al. 2019). Conversely, both protein and mRNA SIRT1 levels decline with age in various tissues of senescence-accelerated mouse prone 8 (SAM-P8) (Braidy et al. 2015; Gong et al. 2014). We conclude that the increase in HSF1 acetylation in Ames wild type hippocampus corresponds to an age-dependent decrease in SIRT1 protein levels. This means that any corrections to the multi-faceted regulation of HSF1 and aging need to address SIRT1 levels and its enzymatic activity.

### HSF1 and protein interactions

In addition to age-dependent post-translational modifications of HSF1, another mechanism for attenuation of HSF1 activity is through protein interactions. Complexes of HSP40-70 and 90 interact with the eukaryotic proteome and modulate the activity of transcription factors including HSF1 (Shi et al. 1998; Akerfelt et al. 2010; Pahlavani et al. 1996; Heydari et al. 1995; Fargnoli et al. 1990). Our previous study in liver tissue from Ames wild type and long-lived Dwarf mice showed that the expression of HSPs (HSP40, HSP70 and HSP90) increase with age in Ames wild type mice while it decreases with age in long-lived Dwarf mice. The results from our current study in brain suggests that HSP levels (HSP40, HSP70 and HSP90) do not reveal a consistent pattern of change to account for loss of HSF1–DNA binding activity. While HSP40 and HSP70 protein levels decline with age in the brain of both Ames wild type and Dwarf long lived mice, Dwarf mice brains have higher levels of HSP90 than Ames wild type mice. Our interpretation is that long-lived mice have an enhanced mechanism of downregulating HSF1 after the stress response because HSP90 has been linked to the conversion of HSF1 trimers to the basal state of HSF1 monomers (Ali et al. 1998). The whole question of heat shock plasticity (i.e., on and off responses over repetitive stress) is not fully understood during senescence. Whether HSP90 levels in long-lived mice brain tissue play a critical role in longevity that is independent of other heat shock proteins is uncertain.

## Summary

The primary conclusion is that brain HSF1 activation is attenuated during normal senescence whereas it is enhanced with exceptional longevity. The mechanism for reduced HSF1 function with normal aging appears to be mostly due to loss of HSF1 protein levels and an increase in its non-functional, acetylated form. Elevated levels of acetylated HSF1 within aging brain suggests that HSF1 degradation is accelerated as opposed to its under synthesis.

From an anti-aging perspective, ramping up SIRT1 levels and its enzymatic activity could have a significant impact on reconstituting the HS axis during aging. Preliminary data with a putative anti-aging compound, resveratrol, show partial enhancement of HSF1 activation (unpublished data).

Another conclusion from this study is that age-dependent changes in the HS axis show organ specific differences, so any anti-aging interventions need to take into account that heat shock enhancers may not produce the same results across all tissue. Signal transduction linked to p38MAPK appears to be key candidate for bolstering HSF1 during aging.

While this study shows the impact of aging and longevity on HSF1 regulation, there are several unresolved issues. Firstly, we do not know the exact time in the lifespan when brain HSF1 function declines, noting it must be sometime between 3 and 12 M of age in mice. The decline is global and not gender specific (unpublished data). However, we note considerable variation between animals and changes in their HS axis, thus raising the possibility that biological rather than chronological aging impacts the HS axis. Also, our immunochemistry analysis of HSF1 protein levels in the hippocampus reveals that the age-dependent declines in HSF1 may be regional if not cell specific.

Finally, changes in HSF1–DNA binding levels with age are detected by *in vitro* assays and may not reflect actual *in vivo *activity related to the heat shock promoter region (Hentze et al. 2016). Some of our recent unpublished experiments suggest that the nGAAn promoter region associated with the heat shock genes is not hypermethylated with aging, thus indicating that any problems with age-dependent heat shock gene expression are most likely via defective HSF1 activation. From a clinical perspective, the brain heat shock response and the impact of aging may be very different for acute events such as traumatic brain injury versus more indolent events such as progressive multi-infarct dementia or Alzheimer’s Disease. Nonetheless, efforts to revitalize the HS response during brain senescence may be a way to prevent or delay neurodegeneration.

### Supplementary Information

Below is the link to the electronic supplementary material.

Supplementary file1 (PPTX 8352 KB)

## Data Availability

Not applicable.
