# Supplementary material for: Disrupted HSF1 regulation in normal and exceptional brain aging
Source: Biogerontology. 2023 Sep 14;25(1):147–60. doi: 10.1007/s10522-023-10063-w (PMC10794279; doi:10.1007/s10522-023-10063-w)
Supplement: Supplementary file 1 — Supplementary file1 (PPTX 8352 KB) [file 10522_2023_10063_MOESM1_ESM.pptx]

## Slide 1
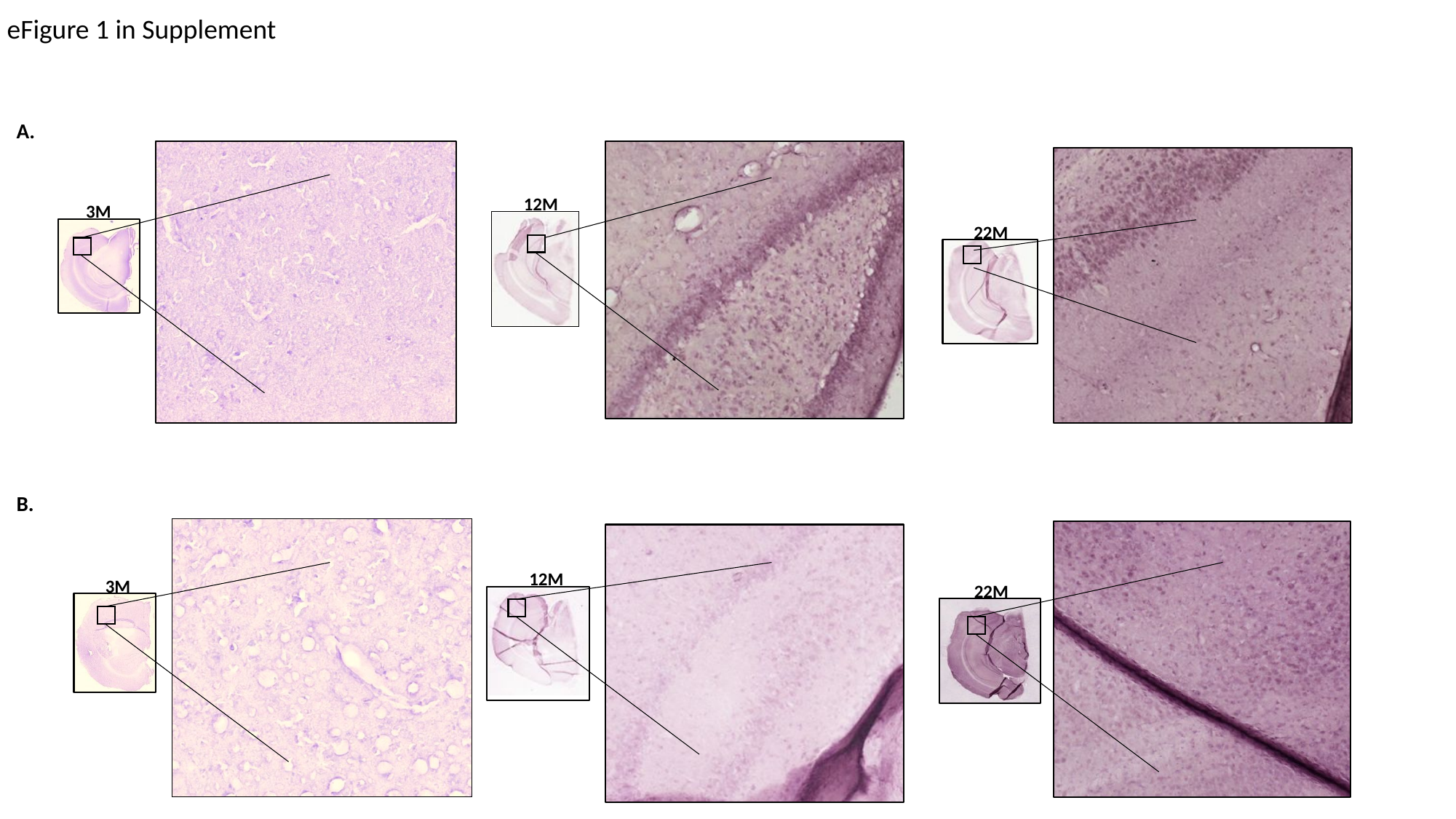

eFigure 1 in Supplement
A.
12M
3M
22M
B.
12M
3M
22M

## Slide 2
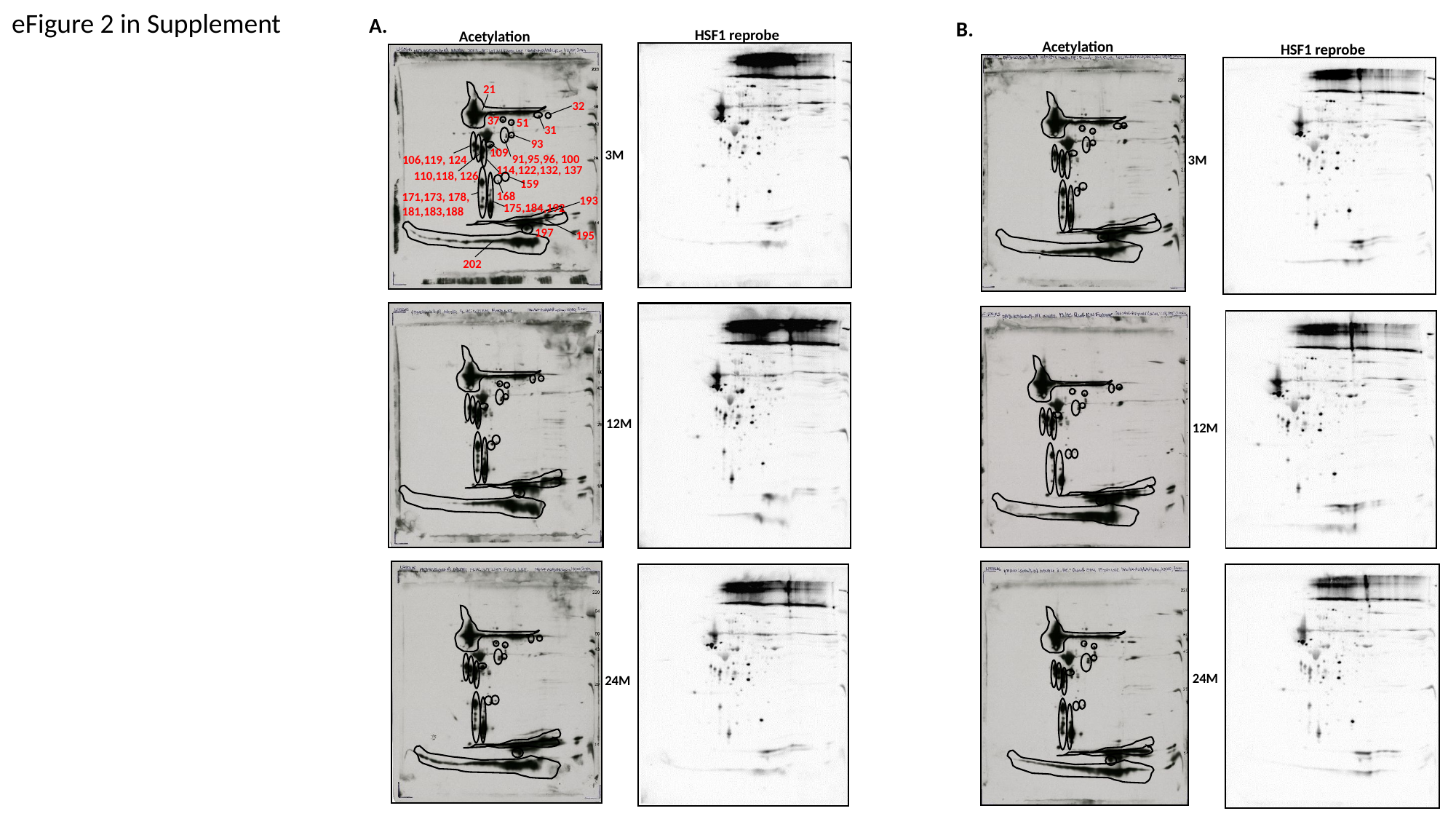

eFigure 2 in Supplement
A.
B.
HSF1 reprobe
Acetylation
Acetylation
HSF1 reprobe
21
32
37
51
31
93
109
3M
91,95,96, 100
106,119, 124
3M
114,122,132, 137
110,118, 126
159
168
171,173, 178,
181,183,188
193
175,184,192
197
195
202
12M
12M
24M
24M

## Slide 3
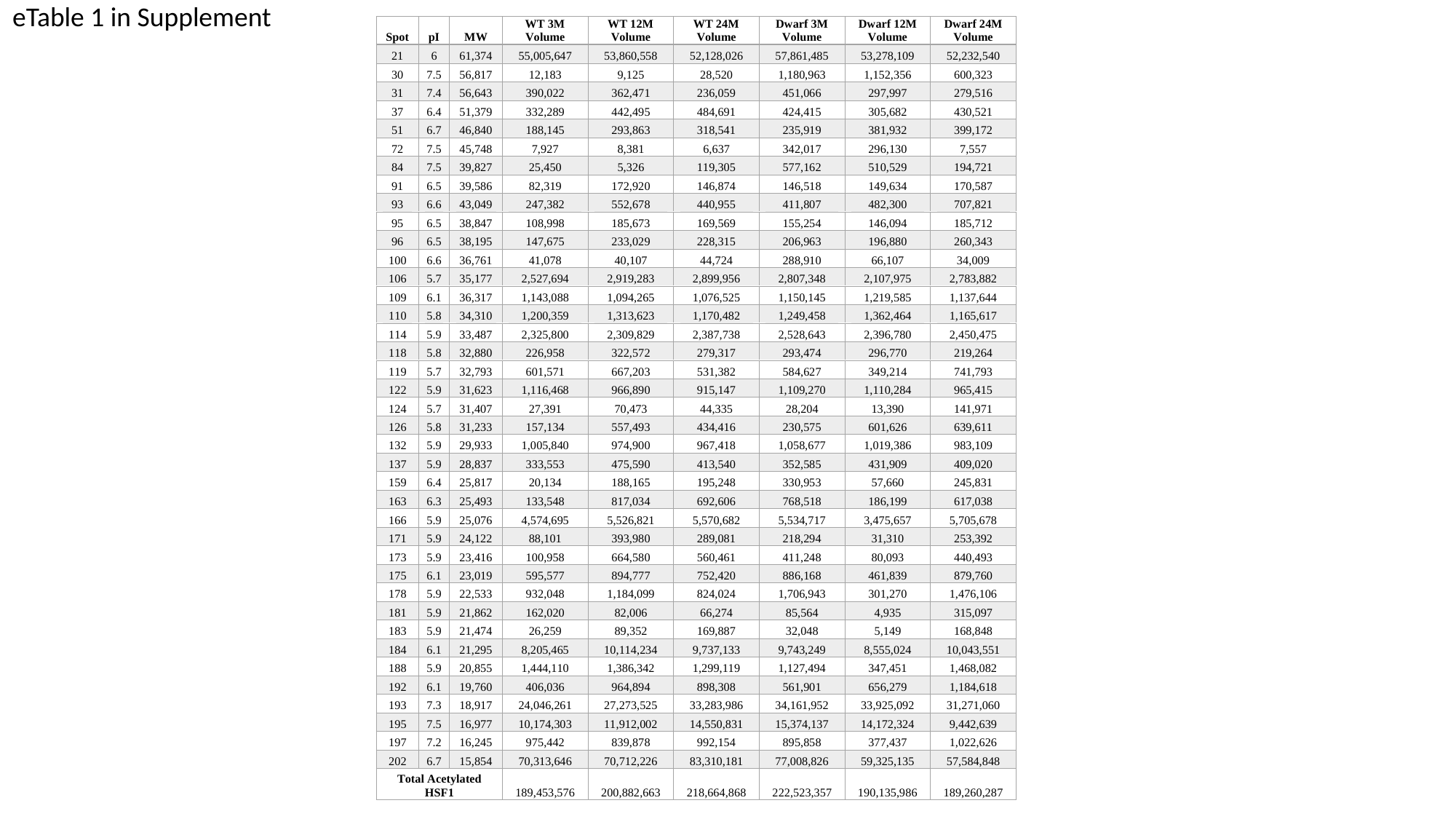

eTable 1 in Supplement

## Slide 4
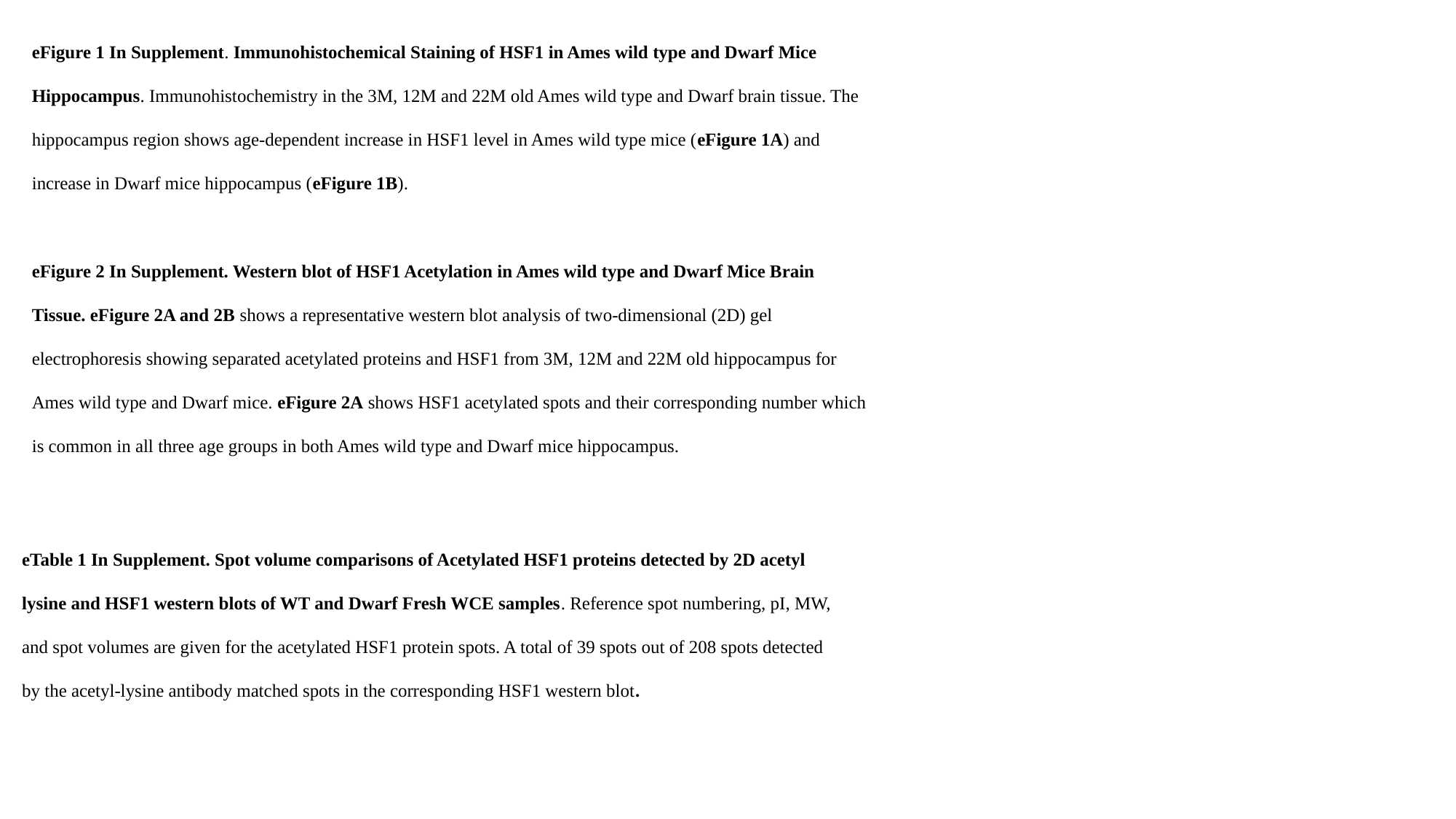

eFigure 1 In Supplement. Immunohistochemical Staining of HSF1 in Ames wild type and Dwarf Mice Hippocampus. Immunohistochemistry in the 3M, 12M and 22M old Ames wild type and Dwarf brain tissue. The hippocampus region shows age-dependent increase in HSF1 level in Ames wild type mice (eFigure 1A) and increase in Dwarf mice hippocampus (eFigure 1B).
eFigure 2 In Supplement. Western blot of HSF1 Acetylation in Ames wild type and Dwarf Mice Brain Tissue. eFigure 2A and 2B shows a representative western blot analysis of two-dimensional (2D) gel electrophoresis showing separated acetylated proteins and HSF1 from 3M, 12M and 22M old hippocampus for Ames wild type and Dwarf mice. eFigure 2A shows HSF1 acetylated spots and their corresponding number which is common in all three age groups in both Ames wild type and Dwarf mice hippocampus.
eTable 1 In Supplement. Spot volume comparisons of Acetylated HSF1 proteins detected by 2D acetyl lysine and HSF1 western blots of WT and Dwarf Fresh WCE samples. Reference spot numbering, pI, MW, and spot volumes are given for the acetylated HSF1 protein spots. A total of 39 spots out of 208 spots detected by the acetyl-lysine antibody matched spots in the corresponding HSF1 western blot.
